# Skin Lesion Classification in Head and Neck Cancers Using Tissue Index Images Derived from Hyperspectral Imaging

**DOI:** 10.3390/cancers17101622

**Published:** 2025-05-11

**Authors:** Doruntina Hoxha, Aljoša Krt, Jošt Stergar, Tadej Tomanič, Aleš Grošelj, Ivan Štajduhar, Gregor Serša, Matija Milanič

**Affiliations:** 1 Faculty of Mathematics and Physics, University of Ljubljana, 1000 Ljubljana, Sloveniatadej.tomanic@fmf.uni-lj.si (T.T.);; 2 Izola General Hospital, 6310 Izola, Slovenia; aljosa.krt@gmail.com; 3 Jožef Stefan Institute, 1000 Ljubljana, Slovenia; 4 Department of Otorhinolaryngology and Cervicofacial Surgery, University Medical Center Ljubljana, 1000 Ljubljana, Slovenia; ales.groselj@mf.uni-lj.si; 5 Faculty of Engineering, University of Rijeka, 51000 Rijeka, Croatia; ivan.stajduhar@uniri.hr; 6 Institute of Oncology Ljubljana, 1000 Ljubljana, Slovenia; gsersa@onko-i.si; 7 Faculty of Health Sciences, University of Ljubljana, 1000 Ljubljana, Slovenia

**Keywords:** hyperspectral imaging, tissue index images, tumors, machine learning

## Abstract

Background: Skin lesions associated with head and neck carcinomas present a diagnostic challenge. Conventional imaging methods, such as dermoscopy and RGB imaging, often face limitations in providing detailed information about skin lesions and accurately differentiating tumor tissue from healthy skin. Methods: This study developed a novel approach utilizing tissue index images derived from hyperspectral imaging (HSI) in combination with machine learning (ML) classifiers to enhance lesion classification. The primary aim was to identify essential features for categorizing tumor, peritumor, and healthy skin regions using both RGB and hyperspectral data. Detailed skin lesion images of 16 patients, comprising 24 lesions, were acquired using HSI. The first- and second-order statistics radiomic features were extracted from both the tissue index images and RGB images, with the minimum redundancy–maximum relevance (mRMR) algorithm used to select the most relevant ones that played an important role in improving classification accuracy and offering insights into the complexities of skin lesion morphology. We assessed the classification accuracy across three scenarios: using only RGB images (Scenario I), only tissue index images (Scenario II), and their combination (Scenario III). Results: The results indicated an accuracy of 87.73% for RGB images alone, which improved to 91.75% for tissue index images. The area under the curve (AUC) for lesion classifications reached 0.85 with RGB images and over 0.94 with tissue index images. Conclusions: These findings underscore the potential of utilizing HSI-derived tissue index images as a method for the non-invasive characterization of tissues and tumor analysis.

## Simple Summary

Our study presents a novel, non-invasive method for classifying skin lesions in head and neck carcinomas. Using machine learning techniques and hyperspectral imaging-derived tissue index images, which are estimations of properties such as blood content, oxygenation, melanin, and water content, we accurately distinguished tumor, peritumor, and healthy skin regions. The results demonstrated strong classification performance, highlighting the potential of tissue index images for non-invasive tissue characterization and tumor analysis.

## 1. Introduction

**Figure 1:**
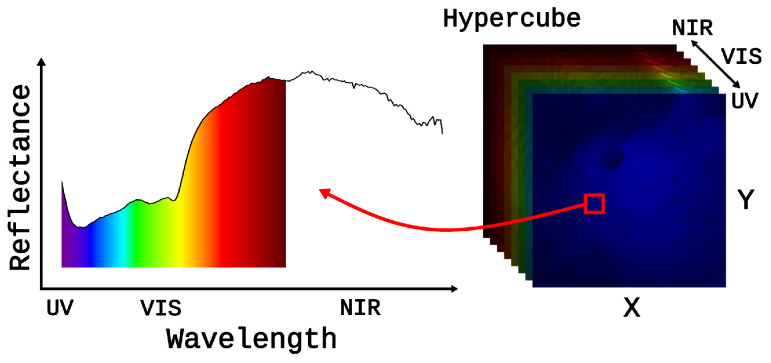
Illustration of a 3D hyperspectral cube, with x and y dimensions indicating the spatial location of the sample, and the third dimension representing the light spectrum captured at each pixel within the image. The red arrow points from a small region within the hyperspectral cube to its corresponding reflectance spectrum, illustrating how spectral data are extracted from that location.

Skin cancer poses a significant global health challenge, as evidenced by its increasing prevalence over the years [1]. The early and accurate diagnosis of skin lesions is critical for effective treatment. Conventional imaging methods, such as dermoscopy [2] and RGB imaging [3], often face limitations in providing detailed information about skin lesions. In response to this challenge, hyperspectral imaging (HSI) emerged as a promising non-invasive and contactless technique [4,5,6], offering a comprehensive view of skin lesions by capturing a broad range of wavelengths. HSI capabilities exceed those of traditional methods, allowing for the extraction of detailed spectral information from the observed tissues. This technique combines imaging and spectroscopy, producing data in the form of a 3D hyperspectral cube where the first two dimensions represent the spatial location of the sample, and the third dimension represents the light spectrum (see Figure 1).

In medical applications, HSI provides images rich in information about the tissue examined, including details about its structure, chemical composition, and other physical properties. This wealth of information assists in diagnosing diseases, monitoring treatment progress, identifying changes in tissue, and providing guidance during surgical procedures. The advanced capabilities of HSI make it a valuable tool for characterizing skin lesions with high precision [4,5,6].

In the past few years, numerous studies have explored the utilization of HSI in detecting skin lesions [7,8,9,10,11]. Various approaches have utilized artificial intelligence (AI) and HSI for precise lesion segmentation and classification [12,13,14,15,16,17,18]. Huang et al. [12] utilized AI and HSI to distinguish mycosis fungoides with high sensitivity and specificity. Nagaoka et al. [13] developed a melanoma screening system using a melanoma discrimination index based on pigment characteristics. Lindholm et al. [14] applied convolutional neural network (CNN) analysis with a hand-held spectral imager to differentiate both pigmented and non-pigmented lesions, achieving notable accuracy. Additionally, Leon et al. [15] differentiated pigmented lesions through a combination of algorithms, while Hosking et al. [16] achieved high sensitivity but low specificity in melanoma detection. Parasca et al. [17] assessed carcinoma margins with spectral angle mapping (SAM), and Courtenay et al. [18] developed classification tools using near-infrared hyperspectral imaging to differentiate between non-melanoma skin cancers and actinic keratosis. A summary of these studies, including their methodology, results, and limitations, is provided in Table 1.

In our study, we leveraged the power of HSI to analyze skin carcinomas of the head and neck regions. We explored the extraction of radiomic features from tissue index images derived from hyperspectral data, providing a unique perspective for analyzing skin lesions. By integrating radiomic features derived from these images with machine learning (ML) techniques, our aim was to uncover the radiomic signatures associated with tissue characteristics, contributing to a deeper understanding of skin lesion pathology. However, the lack of standardized radiomic pipelines presents challenges to ensuring the reproducibility and clinical applicability of findings in the field [19]. Currently, we are at IDEAL stages 2a to 2b, focusing on advancing the application and assessing the feasibility, safety, and potential benefits of our approach with a small number of patients, before moving on to more rigorous evaluations and comparisons with other methods [20]. The aim of this pilot study was to identify the most relevant radiomic features derived from tissue index images to determine which of these indices possessed higher discriminative power, ultimately contributing to higher classification accuracy in differentiating between tumor, peritumor, and healthy skin regions.

Specifically, we conducted a classification analysis in three scenarios: when only RGB images were used (Scenario I), when tissue index images were used (Scenario II), and when their combination was used (Scenario III). This exploration allowed us to discern if the information from tissue index images enhanced the accuracy of distinguishing between tumors, peritumor areas, and healthy skin regions in the head and neck.

Contrasting the existing methods, which dominantly relied on full hyperspectral images, the approach presented in this paper uses biologically significant tissue index images that provide dimensionality reduction while being indicative of the morphological and physiological changes associated with skin carcinoma, allowing for a more comprehensive analysis of tissue characteristics.

Through the extraction and identification of the most relevant radiomic features that are characteristic of the tumor, the presented analysis provides a new layer of interpretability that has not been sufficiently addressed in previous studies. By focusing on the features extracted from tissue index images, which are estimations of blood content, oxygenation, melanin, water content, and other relevant properties, our approach identifies meaningful and interpretable biomarkers that contribute to the overall interpretability of the classification process.

The developed pipeline that integrates radiomic feature extraction, feature selection, and classification allows for the identification and selection of the most relevant radiomic features for more reliable classification.

The evaluation of classification performance across three different scenarios allowed us to assess the effectiveness of the proposed pipeline and the resulting models in different contexts as well as improves our understanding of how each image type contributes to lesion classification.

## 2. Data and Methodology

### 2.1. Imaging System

A handheld hyperspectral camera, Specim IQ (Specim, Oulu, Finland), with a 400–1000 nm spectral range, 7 nm spectral resolution, and 512 × 512 spatial pixels, was used to perform the imaging. The camera was mounted on a photographic tripod, ensuring stability. The field of view covered approximately 10 × 10 cm^2^. Illumination was provided by a 150 W halogen lamp coupled to a ring illuminator through an optical fiber bundle (MI-150DG1, Vision Light Tech, Uden, The Netherlands), ensuring adequate lighting across the entire spectral range of the camera. In order to minimize direct reflections from the skin, a set of crossed polarizers was placed in front of both the camera and the ring illuminator.

### 2.2. Data Acquisition

Skin images captured were normalized using a pre-recorded image of a white standard (Spectralon, Labsphere Inc., North Sutton, NH, USA). Using a white reference designed specifically for such purpose eliminated the need to verify the standard itself. The raw hyperspectral radiance was converted into reflectance using the following equation [4]:

(1) $$I_{\mathrm{ref}}=\frac{I_{\mathrm{raw}}-I_{\mathrm{dark}}}{I_{\mathrm{white}}-I_{\mathrm{dark}}}$$

where $I_{\mathrm{raw}}$ represents the raw hyperspectral intensity, $I_{\mathrm{white}}$ denotes the intensity of the white standard reference, and $I_{\mathrm{dark}}$ is the dark current measured when the camera shutter is closed. After normalization, the images were denoised using the minimum noise fraction (MNF) technique [21]. In brief, MNF first whitens the noise by estimating the noise covariance matrix from neighbouring pixels and applies it to the data, thus removing correlations and effectively making the noise white. Following this step, MNF performs principal component analysis to determine the components containing the majority of the signal. Using only these components the image can then be back-projected into a noise-filtered version.

**Figure 2:**
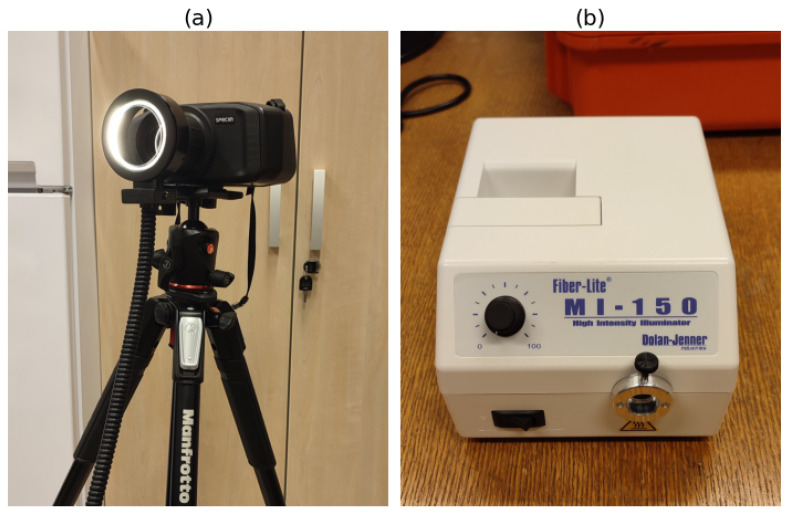
Imaging setup: (**a**) Specim iQ camera mounted on a photographic tripod; (**b**) Fiber-Lite MI-150 high-intensity illuminator.

The imaging itself was performed in a few distinct steps. Before starting this work, the camera was adequately prepared by fully charging the batteries and verifying the calibration, as per the manufacturer’s instructions, which included imaging an orange sample provided with the camera possessing known reflectance values and comparing the results to the on-camera reference measurement. The whole process was automated in the camera control software. Before the acquisition itself, the illumination light source was warmed up to reduce the effects of heating. The position of crossed polarizers in front of the objective and on the ring light used for the illumination was verified with a metallic mirror and adjusted as necessary to achieve minimal mirror reflection. Following this step, the procedure room was blacked out by turning off the ambient lights and closing the window shades, if applicable. Following the preparation, just before imaging the patient, a white reference was acquired using the standard protocol for the camera by placing the reference standard at a predefined distance using a height standard. The distance from the camera to the sample was set in such a way that the white reference was imaged at the same distance as the lesion itself, where the overall surfaces were highly curved. The imaging of the patients themselves was conducted by first covering their eyes and then setting them at an appropriate distance from the camera, defined by the height standard. Figure 2 shows the camera setup used during the imaging process, along with the Fiber-Lite MI-150 high-intensity illuminator, which ensured adequate lighting conditions.

To minimize any potential movement during the measurements, the patients were lying down on an examination bed while the images were captured. To achieve good positioning, motorized height control of the exam table was used. During the acquisition, the same integration time for the acquisition of the white reference and the image itself was assured to both prevent oversaturation as well as eliminate possible uncertainties introduced by the on-board normalization of the data to the exposure time. Typical values for the integration time were 50 ms per line, thus resulting in a total acquisition time of about 30 s for the whole image. Following the image acquisition, some preliminary quality assurance on the data was performed by the operator; the image was checked for saturation and appropriate spectral shapes of the normalized reflectance in the tumor area and in the surrounding tissue by validating the general trends (hemoglobin absorption peaks) and absolute values (must be lower than 1, preferably between 0.3 and 0.7). Finally, the light source was turned off, and the normal clinical workflow was resumed. Before using the equipment, the protocol was tested, and new operators were thoroughly trained by an experienced user of the imaging system.

### 2.3. Tumor Dataset Overview

The dataset in this study contained 24 head and neck skin carcinomas from 16 patients, including BCC and SCC types that were selected clinically for ECT treatment. Among these patients, there were 6 women and 10 men, with an age range of 60 to 95 years. These cases were recruited from the Department of Otorhinolaryngology and Cervicofacial Surgery, Ljubljana. Clinical and histopathological characteristics, including patient age, sex, tumor location, and histopathological type (SCC or BCC), were recorded for each participant. A summary table of these data is provided in Appendix A.

Patients were selected for ECT based on several inclusion criteria: histologically or cytologically confirmed squamous cell or basal cell carcinoma in the head and neck area, the unsuitability of tumors for standard treatment (due to factors like tumor location or patient refusal), being over 18 years old, and maintaining a Karnofsky [22] performance status of 70 or higher. Key exclusion criteria included life-threatening systemic diseases, severe coagulation disorders, and significantly impaired lung function. For a detailed list of inclusion and exclusion criteria, please refer to Appendix B.

The dataset consisted of RGB images and 12 tissue index images derived from raw hyperspectral data, which were opened and processed using MATLAB (Mathworks, Natick, MA, USA). To generate the RGB images, normalized spectra were projected on the CIE XYZ colorspace by multiplication with the tristimulus values and converting from XYZ to RGB colorspace using the xyz2rgb function in MATLAB with the standard D65 illuminant option [23,24]. Tissue indices are image metrics or mathematical representations used to evaluate tissue properties based on the reflected light spectra captured by the hyperspectral imaging system [25,26,27,28,29]. These indices were derived from specific spectral bands chosen for their sensitivity to particular tissue characteristics or physiological parameters. The tissue indices used for this analysis are given in Table 2, and the equations for calculating these indices are provided in Appendix C.

This study’s protocol aligned with the guidelines outlined in the Declaration of Helsinki and was approved by the Slovenian National Medical Ethics Committee (application number 0120-135/2021/3). Prior to imaging procedures, informed consent was obtained from all human patients participating in this study.

### 2.4. Ground Truth Mask Generation

In addition to the images of the skin carcinomas, the dataset included tumor and peritumor region masks. The peritumor region refers to the area exhibiting visual deviations from normal skin morphology, as assessed through clinical examination, while remaining distinct from the macroscopically defined tumor tissue, which we treat with ECT electrodes. This area is clinically relevant because it allows the effective targeting of adjacent tissue that may be affected by the tumor, ensuring comprehensive treatment.

The masks were segmented collaboratively and simultaneously by two medical experts. The initial assessment was conducted through clinical examination, after which the experts delineated tumor and peritumor borders on the RGB photos. Both experts worked together throughout the entire segmentation process, discussing and refining the tumor boundaries in real time to ensure clinical accuracy. A single mask was generated for each image, which was then used for all subsequent analyses.

Furthermore, after delineating the tumor and peritumoral regions, the entire skin region was separated from the background using image processing techniques like the spectral angle mapper (SAM) [30]. The SAM evaluates the spectral resemblance between the spectra in each pixel of a hyperspectral image and a designated reference spectrum to distinguish the skin from the background. The reference spectrum was derived by manually selecting a pixel in the skin region from an RGB image to identify its coordinates, which were then used to extract the corresponding values from all the other tissue indices derived from the hyperspectral images. This process provided us with a reference spectrum for the skin region and was repeated for each image in the dataset. This technique allowed us to distinguish the skin region from the surrounding elements, effectively eliminating unwanted background.

These regions formed the foundation for our classification task, representing the three main classes in our study. In total, we had 72 regions of interest, with 24 representing tumors (class I), 24 representing peritumor regions (class II), and another 24 representing the healthy regions of the skin (class III), each providing insights into the diverse tissue types under investigation.

**Figure 3:**
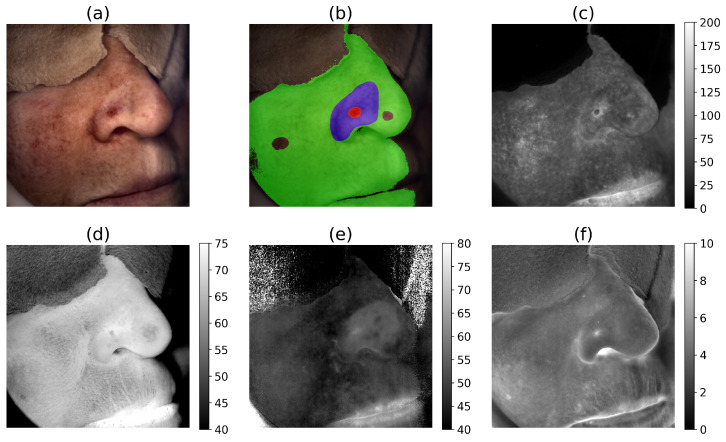
A volunteer with a BCC tumor on the nose. (**a**) RGB image showing the patient’s head. (**b**) Masked overlay on the RGB image, indicating the regions of interest. Red corresponds to the tumor, blue represents the peritumor region, and green denotes healthy skin. Two rounded areas within the green healthy skin regions were excluded from the analysis due to the presence of benign tumors. Grayscale images show tissue indices along with the color bars indicating their range values: (**c**) Dawson’s erythema index, (**d**) Ishimaru’s oxygenation index, (**e**) Huang’s oxygenation index, and (**f**) Dawson’s melanin index.

Figure 3 shows a volunteer with a BCC tumor on the nose. The quantitative comparison of the tissue indices in the tumor, peritumor, and healthy tissues is shown in the grayscale subfigures presented in Figure 3, each accompanied by a color bar indicating the corresponding index values. Figure 3a shows the RGB image of a volunteer’s head, with the corresponding masked overlay presented in Figure 3b, highlighting three regions of interest: the tumor region in red, the peritumor region in blue, and the healthy skin in green. The erythema index (Figure 3c) exhibited an average value of 98.97 ± 21.43 for the tumor, 70.58 ± 13.32 in the peritumor region, and 67.87 ± 20.70 for the healthy skin. These values suggested increased blood concentration in the tumor region and decreased levels in healthy regions. Deeper skin oxygenation, as indicated by Ishimaru’s oxygenation index (Figure 3d), exhibited higher values in tumor regions, with values of 70.16 ± 2.12, compared to 68.41 ± 2.24 in the peritumor and 65.34 ± 4.02 in the healthy skin. Similarly, superficial oxygenation, represented by Huang’s oxygenation index (Figure 3e), also demonstrated elevated values in tumor regions compared to the peritumor area and the healthy skin. Additionally, the melanin index (Figure 3f) revealed differences between the tumor and healthy skin regions, with values of 5.92 ± 0.94 in the tumor and 4.46 ± 0.91 in the healthy skin, further emphasizing the variations observed in tissue characteristics across different regions.

### 2.5. Feature Extraction and Selection

PyRadiomics Python library (version 3.1.0) [31] was utilized to extract first- and second-order radiomic features from both the RGB images and 12 tissue index images for each region. PyRadiomics provided a wide range of features, generally classified as shape-based and first- and second-order statistics. For a comprehensive overview of these features, please refer to Scapicchio et al. [32], which includes detailed descriptions of the classes of features, such as first-order and second-order statistics, that were used in this analysis.

Independent of the gray-level intensity distribution within the region of interest, the shape-based features describe the 2D or 3D size and shape of the region of interest. This feature class was intentionally excluded during the initial extraction process, considering the unique morphological attributes of the tumor, peritumor, and healthy skin regions. Given the distinct shapes—tumors being round, peritumors being round with a central void, and healthy skin being square with a central void—incorporating shape features was deemed unnecessary. This enabled us to concentrate on extracting features that provided greater insight into the tissue composition and pathology, thereby enhancing the precision of our classification.

First-order statistics features, such as energy, standard deviation, and entropy, quantify the distribution of the pixel intensity values within a given region of interest. These features assess pixel values independent of their spatial arrangement, providing insights into the overall distribution of intensity values within the image. For more detailed definitions, including the equations for the calculation of these features as well as the second-order features, please refer to the PyRadiomics documentation [31], which served as a reference standard for the radiomic analysis.

Second-order statistics features focus on how pairs of pixels are distributed spatially, revealing patterns not easily seen by the human eye. In our analysis, we used these features as the input for our classifiers to improve the differentiation between tumoral and healthy tissue by leveraging the textural information they provided.

The analysis of second-order statistics features involved two steps: first, creating a matrix that captured the spatial distribution of pixel values; second, calculating metrics from this matrix. Once the matrix was created, metrics were computed to summarize the information contained within it. Each matrix produced a single value for each feature, reflecting the spatial relationships and distributions of the pixel intensities between pairs of pixels.

Common matrices used include the Gray-Level Co-occurrence Matrix (GLCM), Gray-Level Run Length Matrix (GLRLM), Gray-Level Size Zone Matrix (GLSZM), Neighboring Gray-Tone Difference Matrix (NGTDM), and Gray-Level Dependence Matrix (GLDM). The GLCM contains statistical information that represents the distribution of pixel pairs throughout the image. The length of successive pixels in an image with the same intensity in a predetermined direction is expressed by the GLRLM. Gray-level zones, or the number of connected pixels with the same gray-level intensity, are quantified in an image by the GLSZM. The NGTDM measures the difference between the gray value of a pixel and the average gray value of its neighboring pixels within a specified distance. Gray-level dependencies, or the number of connected pixels within a certain distance that are dependent on the center pixel, are quantified with the GLDM in images.

The features of all classes, except shape-based ones, were extracted, resulting in a set of 93 features; however, only those showing a high correlation with the target label were used for the analysis. The selection of the most relevant features was conducted using the minimum redundancy–maximum relevance (mRMR) algorithm [33]. This algorithm employs two criteria, as implied by its name: maximum relevance and minimum redundancy. Maximum relevance ensures the selected features have a strong correlation with the target variable, while minimum redundancy ensures that these features are unique and dissimilar. Initially, we applied the mRMR algorithm to each of the 12 tissue index images, selecting the 7 most relevant features. The same process was repeated for the RGB images, picking out the 7 most relevant features.

Our approach ensured the identification of the key features from each of the 12 tissue index images and RGB images, resulting in an initial set of features. We again utilized the mRMR algorithm to select the 7 most crucial features from the remaining set. This selection process occurred in two scenarios: when using only tissue indices for the analysis and when combining them with RGB images. We chose to test the combination of features from the RGB and tissue index images to evaluate whether the combined approach would yield improved classification results and to determine which type of images provided more relevant information in this case. Our analysis revealed that only one feature from the RGB images was significant in this case, while the majority of the discriminative power came from the features extracted from the tissue index images, underscoring their predominant role in enhancing the overall classification of skin lesions. During our experiments, we explored both lower and higher numbers of features. We found that using fewer features still gave good performance with the tissue index images, achieving AUC values above 0.90, while the results were less favorable for the RGB images. Similarly, increasing the number of features did not lead to any significant improvement in classification accuracy. Ultimately, the combination of 7 features proved to be the best choice. Therefore, we opted to maintain a concise set of features to enhance computational efficiency and avoid overfitting.

While the mRMR algorithm primarily prioritized features that distinguished between tumor and healthy skin due to their more significant differences in feature values, further analysis revealed that certain features were particularly effective in distinguishing between the tumor and peritumor classes. This observation was especially evident in binary classification scenarios where only tumor and peritumor classes were involved. Therefore, in each scenario—Scenario I (using only RGB images), Scenario II (using only tissue index images), and Scenario III (using their combination)—we employed the mRMR algorithm to identify at least two features that demonstrated high discriminatory power between the tumor and peritumor classes. This approach ensured that our final set of features captured important distinctions between tumor and peritumor regions, despite the bias of the mRMR algorithm towards features distinguishing between tumor and healthy skin. This refined selection, leveraging the mRMR algorithm once again, played a key role in optimizing our analysis.

The selected radiomic features for each scenario are summarized in Table 3. While the features from the RGB images in Scenario I are listed only by their names, in Scenarios II and III, the selected features are accompanied by symbols. These symbols (given in Table 2) indicate their origin, specifying from which tissue indices the features were derived. In Scenario III, the symbol “RGB” denotes the feature derived from RGB images.

As shown in Table 3, among the tissue indices, the erythema, oxygenation, and water content indices demonstrated higher discriminative power for the classification analysis, as the features derived from these indices were identified as the most relevant for our analysis.

### 2.6. Automated Model Selection and Hyperparameter Optimization

We utilized the Tree-Based Pipeline Optimization Tool (TPOT) [34,35,36] for model selection and hyperparameter optimization. In the context of ML, hyperparameters are configuration settings that are external to the model and are not learned from the data. They play a crucial role in determining the behavior and performance of the model [37].

The TPOT, an automated ML tool based on genetic programming, systematically explores a range of hyperparameter settings and considers a diverse set of classifiers, including decision trees, support vector machines, and gradient boosting classifiers, during its optimization process. This process enabled us to identify the most suitable model for our dataset with fine-tuned hyperparameters. Hyperparameter optimization is significant as it can considerably impact model performance. By fine-tuning these parameters, we enhanced accuracy, reduced overfitting, and improved the model’s ability to perform better on unseen data. In each of the three scenarios, the TPOT generated a pipeline tailored to the specific data. In Scenario I, the selected 7 radiomic features from RGB images were utilized as the input data for the classification task, which aimed to distinguish between tumor, peritumor, and healthy regions. In this case, TPOT generated the Gradient Boosting Classifier as the most optimal classifier. In Scenario II, the selected 7 features from the tissue indices, such as the Dawson’s erythema index and Huang’s oxygenation index, were used for the classification task. In this scenario, the Extra Trees Classifier was generated by the TPOT as the best-performing model. A pipeline with a unique configuration was generated by the TPOT in Scenario III, which combined features from tissue indices and RGB images. This pipeline included a Stacking Estimator using the SGD Classifier as its foundation, followed by a feature constructor, Zero Count, which counted the occurrences of zero/non-zeros per sample, and concluded with a Linear SVC. The specific hyperparameters for each model are detailed in Table 4. For a detailed description of these hyperparameters, refer to the scikit-learn documentation [38], which provides comprehensive explanations for each parameter used in the classifiers we applied.

Maintaining consistency in our approach, specifically by setting the number of generations to 100 and the population size to 50 in the TPOT, ensured a thorough exploration of the hyperparameter options, aiming for optimal model performance. This approach with the TPOT for model selection and hyperparameter optimization ensured a comprehensive and optimized modeling process. Moreover, for all scenarios, whether using only RGB images, tissue indices, or their combinations, we split our data into 75% for training the classifiers and 25% for testing.

Following this, the best pipelines generated by the TPOT in each of the three scenarios underwent evaluation through repeated stratified k-fold cross-validation. This validation method involved 8 splits and 30 repetitions, ensuring a robust assessment of the model’s generalization performance. To comprehensively evaluate the performance of the generated pipelines, we utilized a set of diverse metrics, including accuracy, precision, recall, balanced accuracy, and F1 score. These metrics were selected to provide an evaluation of the capabilities of models in capturing different aspects of classification performance. The computation of the standard deviations for each metric provided insights into the stability and consistency of the model’s performance across different folds and repetitions.

As a further step in our analysis, we also calculated the Receiver Operating Characteristic (ROC) curves and the AUC as the mean of the four folds for each of the best-performing models using stratified k-fold cross-validation. Utilizing a one-vs.-rest approach to handle the three-class classification problem, the optimal value of k was determined to be 4. For Scenario I, the probability thresholds that yielded optimal performance were found to be between 0.10 and 1.00. In Scenario II, the optimal probability thresholds ranged from 0.10 to 0.90; however, changing the threshold to 0.80 or even 1.00 did not affect the model’s performance. In Scenario III, due to the unique pipeline configuration that did not provide probability outputs, performance metrics such as accuracy, precision, recall, and F1 score, which had been evaluated across all scenarios, were deemed more suitable for evaluating classifier performance. These metrics offered insights into the classification capabilities of the model given its non-probabilistic output nature.

**Figure 4:**
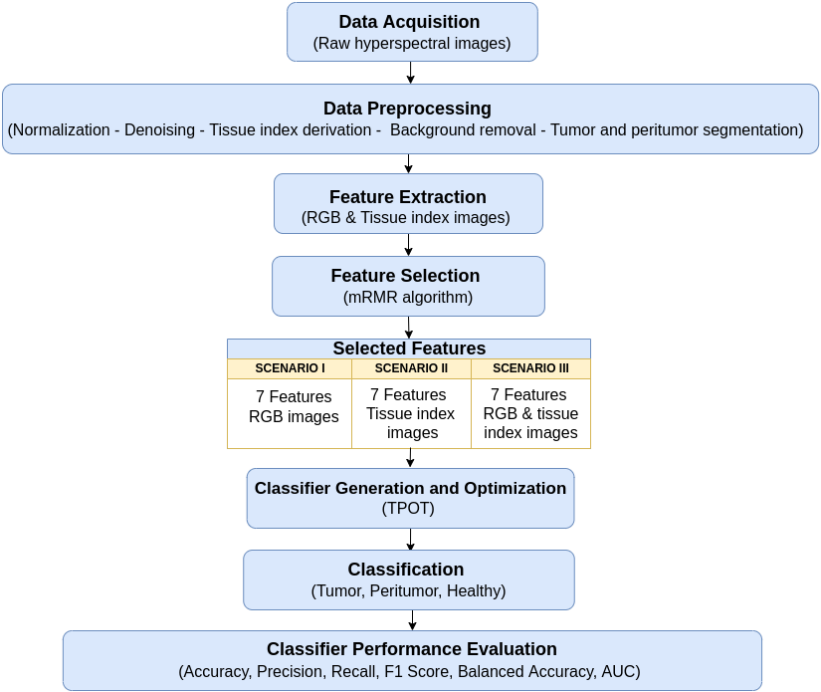
Schematic overview of the workflow.

After generating pipelines using the TPOT, we assessed the importance scores of the features in each scenario. This post-TPOT analysis offered insights into the contribution of individual features to the optimized models. In each case study, whether focusing on RGB images, tissue index images, or their combination, we employed the Extra Trees Classifier to calculate the importance scores. In Figure 4, we present a schematic overview of the workflow.

## 3. Results

### 3.1. Feature Selection

**Figure 5:**
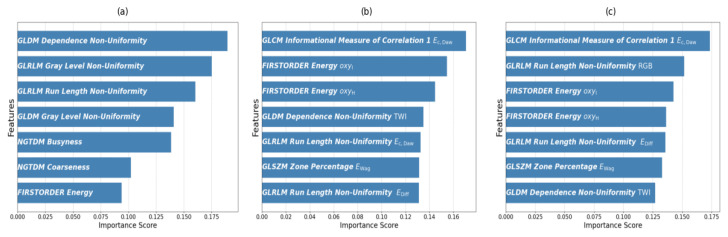
Importance scores of the most relevant radiomic features selected via the mRMR algorithm in three scenarios: (**a**) analysis using features extracted from RGB images, (**b**) analysis employing features derived from tissue index images, and (**c**) combined analysis of both RGB and tissue index images. Each bar represents the importance score of a specific feature, indicating its contribution to the classification of tumor, peritumor, and healthy skin regions.

Initially, our analysis focused on the RGB images, aiming to evaluate classifier performance using the features extracted from this dataset (Scenario I). The mRMR algorithm identified a subset of the most relevant features for understanding the complexities of skin lesion characteristics. The importance scores of the selected features are shown in Figure 5a, with each feature belonging to specific feature groups, including NGTDM, GLRLM, first-order statistics, and GLDM.

In the subsequent phase of our analysis, we exclusively focused on the tissue index images (Scenario II) to explore their potential in distinguishing among distinct classes of skin lesions, including tumors, peritumors, and healthy skin regions. Our aim was to identify the most relevant features for discriminating between these regions of interest. This comprehensive approach allowed us to capture various aspects of skin lesion characteristics related to erythema, total water concentration, superficial oxygenation, and deeper oxygenation, among other tissue indices. Specific features from these indices were distinguished as the most significant. While no features from the GLCM group were prominent in the analysis of the RGB images, a specific feature, ’Imc1- Informational Measure of Correlation 1 $E_{c,\mathrm{Daw}}$’, from this class emerged as a significant contributor in this scenario. Additionally, a feature, ’Zone Percentage’, from the GLSZM group also emerged as a significant contributor in this tissue index analysis, indicating the relevance of this feature group, which was not prominent in Scenario I.

The significance of the selected features is visually depicted in Figure 5b, showing their respective importance scores. The erythema index, reflecting skin redness, and the total water concentration index, indicative of tissue hydration, provided crucial information about the superficial characteristics of lesions. Simultaneously, the superficial oxygenation index and deep oxygenation index, assessing oxygen levels at different depths, offered insights into the vascular and oxygenation aspects of the lesions.

Furthermore, our analysis extended to RGB images combined with tissue index images. The subsequent identification of the seven most relevant features from this combined set is visually represented in Figure 5c, showcasing their importance scores in this scenario. Several features from Scenario II remained significant. This underscored their importance across different contexts. Of particular interest was that among the features selected for this combined approach, only one originated from the RGB images, highlighting the predominant contribution of information from the tissue index images to our analysis. This finding highlights the greater discriminative capability of the tissue index features, demonstrating how important they are for improving classification accuracy and providing deeper insight into skin lesions.

### 3.2. Classifier Performance Using Selected Features

In this subsection, we present the results obtained from the evaluation of the classifiers based on the selected features in three scenarios.

In Scenario I, the optimized pipeline, Gradient Boosting Classifier, demonstrated an accuracy of 87.73%, illustrating its effectiveness in distinguishing between the three classes: tumor (Class I), peritumor (Class II), and healthy skin (Class III). Precision, measuring the model’s ability to avoid false positives, attained a value of 87.84%, while recall reached 87.73%. The F1 score, a balanced metric considering both precision and recall, achieved a value of 87.69%. Additionally, the balanced accuracy, which considers the sensitivity and specificity of the model, attained a value of 87.73%.

For Scenario II, the Extra Trees Classifier achieved an accuracy of 91.76%, precision of 91.89%, recall of 91.76%, F1 score of 91.74%, and balanced accuracy of 91.76%. These results emphasize the effectiveness of using only tissue indices to distinguish tumor, peritumor, and healthy skin classes.

In Scenario III, the TPOT generated a distinctive pipeline configuration, leading to a model showcasing the following performance after repeated stratified k-fold cross-validation: a mean accuracy of 92.04%, mean precision of 92.37% for all classes, mean recall 92.04% for all classes, mean F1 score 92.09% for all classes, and mean balanced accuracy of 92.04%.

**Figure 6:**
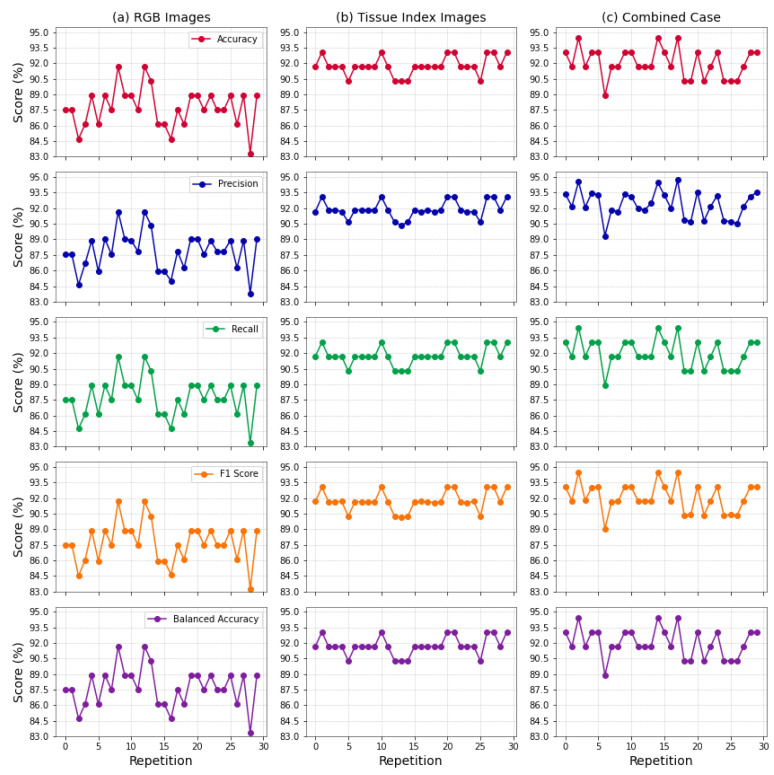
Variation in performance metrics across scenarios. Line plots showing the fluctuation in accuracy, precision, recall, F1 score, and balanced accuracy over 30 repetitions for three scenarios: (**a**) RGB Images, (**b**) tissue index images, and (**c**) combined case.

In summary, Table 5 provides a concise comparison of the key performance metrics across diverse scenarios, offering mean values and their corresponding standard deviations. The metrics—accuracy, precision, recall, F1 score, and balanced accuracy—provide a comprehensive evaluation of model effectiveness in scenarios utilizing RGB images, tissue index images, and a combination of both. In addition to the mean and standard deviation comparison provided in Table 5, we present a visual representation of the performance metrics across multiple repetitions for each scenario. Figure 6 showcases the variability in the key metrics for (a) Scenario I, (b) Scenario II, and (c) Scenario III. Each line in the plots represents the performance of the respective metric over 30 repetitions, providing insights into the consistency of and fluctuations in model performance. The detailed figures enable a more nuanced understanding of the stability and reliability of the generated pipelines in the different scenarios. In Scenario I, where only RGB images were used, the observed variation averaged around 9.50%, indicating notable fluctuations. This variability highlights the dynamic nature of the pipeline’s performance over repeated experiments. Conversely, in Scenario II, which focused on tissue index images, a much lower variation of approximately 3.03% was observed, suggesting greater stability in performance. In Scenario III, where RGB and tissue index images were analyzed together, intermediate variation percentages of about 6.04% were obtained.

**Figure 7:**
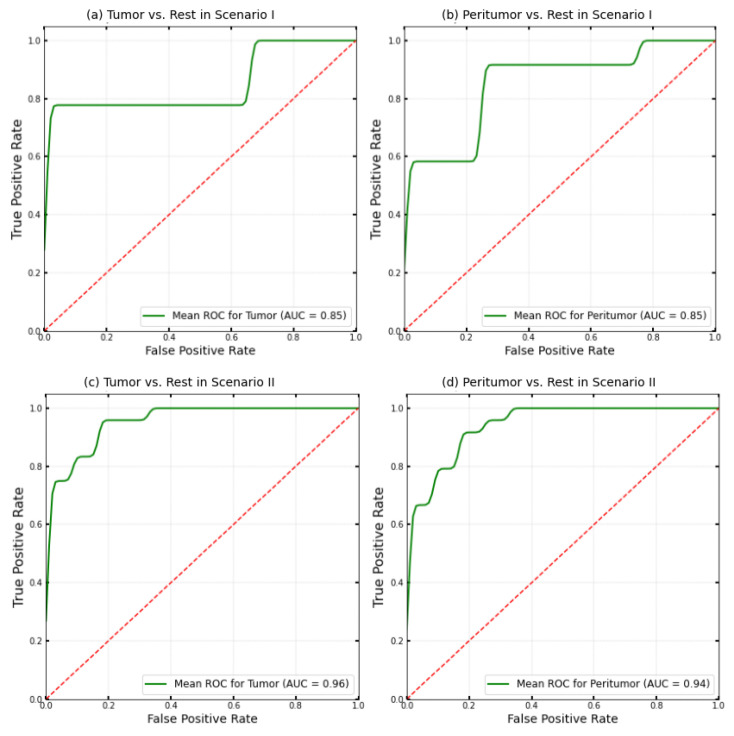
Classification performance across scenarios. ROC curves depicting the ability to discriminate between tumor and peritumor classes in different scenarios: (**a**) tumor vs. rest in Scenario I (AUC = 0.85), (**b**) peritumor vs. rest in Scenario I (AUC = 0.85), (**c**) tumor vs. rest in Scenario II (AUC = 0.96), and (**d**) peritumor vs. rest in Scenario II (AUC = 0.94). The dashed red line in all subfigures serves as the reference line, since it is the ROC curve of random classification with an AUC of 0.5.

To further evaluate the performance of the classifiers in Scenarios I and II, ROC curves were generated for each class, but only the classifications of tumor vs. rest and peritumor vs. rest are shown in Figure 7. These curves illustrate the relationship between the true positive rate (sensitivity) and the false positive rate (1 – specificity) across different classification probability thresholds, with results averaged over a stratified 4-fold cross-validation to ensure robustness.

The ROC curves for tumor vs. the rest classification (Figure 7a for Scenario I and Figure 7c for Scenario II) demonstrate differences in classifier performance between the two scenarios. In Scenario I, the ROC curve yielded an AUC of 0.85 and showed a plateau where a constant true positive rate of 0.79 was maintained, while moving leftward to lower false positive rates. This indicated that as the probability threshold decreased, the model consistently identified tumor cases with fewer misclassifications. Scenario II achieved an AUC of 0.96 for the tumor vs. rest classification, reflecting superior discriminative ability when using tissue index images. The ROC curve is positioned closer to the top left corner, indicating a higher rate of true positives and a lower rate of false positives across various probability thresholds.

The ROC curves for peritumor vs. the rest classification (Figure 7b for Scenario I and Figure 7d for Scenario II) reveal similar differences in classifier performance as observed in the tumor vs. the rest classification. Scenario II showed improved performance with an AUC of 0.94, compared to 0.85 in Scenario I. The ROC curve for the “healthy vs. rest” classification is not presented here, as both scenarios yielded an AUC of one, showing that the classifiers perfectly identified healthy tissues. These AUCs aligned with the recall of 1.0000 for Class III (healthy regions) in both scenarios. While these high metrics indicated effective identification, they may also have been affected by biases associated with the extracted features related to region size.

Overall, the findings highlight that utilizing tissue index images in Scenario II significantly improves the classifier’s ability to identify tumor and peritumor tissues accurately, as evidenced by higher AUCs.

## 4. Discussion

The integration of HSI with ML techniques offers advantages in the analysis of skin lesions. By capturing both spatial and spectral information, HSI provides rich data that can reveal the subtle variations in tissue properties associated with different lesion types. In this study, we investigated the potential of integrating HSI-derived tissue indices with ML classifiers to enhance the classification of skin lesions, particularly focusing on skin carcinomas in the head and neck region. Our findings demonstrate the promising utility of this approach in improving the accuracy and efficacy of lesion classification.

Moreover, this study highlights the importance of feature selection in maximizing the discriminative power of RGB and tissue index images, where a subset of the most relevant features was identified using the mRMR algorithm. The selected features provided insights into characteristics and patterns associated with skin carcinomas in the head and neck region. Features related to texture, non-uniformity, and energy emerged as significant contributors to the classification task, reflecting the complex nature of skin lesion morphology and composition.

In Scenario I, key features such as GLRLM Gray-Level Non-Uniformity, GLRLM Run Length Non-Uniformity, GLDM Gray-Level Non-Uniformity, and GLDM Dependence Non-Uniformity had higher importance scores. These features capture the non-uniformity in gray-level intensity values and run lengths throughout the image. We found that these values were consistently higher in the healthy skin region than in both peritumor and tumor regions, aligning with our expectation that healthy tissue displays a broader range of grayscale intensities. In addition to these features, the analysis also included features from the NGTDM class, such as busyness, coarseness, and first-order energy, which contributed to the classification but had lower importance scores.

In Scenario II, a feature from the GLCM class, IMC1, stood out as the most significant, with the highest importance score. Extracted from the erythema index image, IMC1 provided insights into texture complexity. Higher negative IMC1 values in tumor regions suggest decreased texture complexity, reflecting the homogeneous nature of tumor tissue. In contrast, lower negative IMC1 values in peritumor and healthy skin regions indicate more complex patterns, consistent with the expected diversity of healthy skin. The analysis also highlighted the significance of first-order energy features extracted from the superficial and deeper oxygenation indices, which were anticipated to be relevant for classification due to the observed higher levels of oxygenation in tumor regions compared to the other areas.

Generally, tumor tissues exhibit lower oxygenation levels due to hypoxia, a common characteristic of cancerous tissues [39]. Our results show an increase in oxygenation, which at first appears as a contradiction to the expected hypoxic state. This is to be expected because HSI gives no depth information, and thus superficial changes and changes at the tumor edge also contribute to the measured data. In these regions, the oxygenation can in fact increase due to the angiogenesis caused by the higher metabolic requirements of the tumor [40].

Furthermore, several other texture features extracted from the tissue water index and three different erythema indices proved relevant in Scenario II. Across these features, higher values were consistently observed in the healthy skin region compared to the tumor regions, indicating greater non-uniformity in healthy skin texture.

In Scenario III, only one feature from the RGB images emerged as the most relevant, highlighting the predominant contribution of tissue index images to our analysis. The consistent significance of several features from Scenario II reinforces their relevance across different contexts. Overall, our findings emphasize the pivotal role of tissue-index-derived features in biomedical image analysis, suggesting their potential as robust biomarkers for tissue classification.

Expanding on the role of tissue-index-derived features in classification, it is important to consider their broader implications for biomedical imaging technology. By identifying the most relevant tissue indices, we gain insight into the spectral bands that contribute most to tissue classification, potentially reducing the cost of HSI devices by focusing on key spectral ranges. Thus, understanding the significance of these indices improves classification accuracy, informs decisions about spectral band selection, and optimizes the efficiency and cost of imaging devices.

The performance of different classification models further validates the efficacy of our approach. Table 5 provides a detailed comparison of the performance metrics across the three distinct scenarios. Each scenario was evaluated using the TPOT, which generated distinct pipelines for optimal model performance. In the scenario using only RGB images, the classifiers achieved a mean accuracy of 0.8773 ± 0.0186, with a mean precision of 0.8784, indicating the classifiers’ ability to avoid false positives. However, there was a slight decrease in precision for Class I compared to Class II and Class III.

When using only tissue index images, both accuracy and precision improved, with mean values of 0.9175 and 0.9189, respectively. This suggests that tissue index images provide valuable information for distinguishing between different classes, resulting in higher classification accuracy.

The mean recall values across all scenarios aligned with the mean accuracy, reflecting the classifiers’ ability to correctly identify positive instances. In scenarios using the RGB images and tissue index images individually, Class III achieved a perfect recall of 1.0000. However, this perfect recall may have been influenced by bias from the extracted features related to region size. Similarly, in Scenario III, where RGB and tissue index images were combined, Class III also achieved perfect precision of 1.0000. This outcome could also be attributed to the same factors related to the extracted features.

Additionally, considering that the total pixel count for the healthy skin regions in our analysis was higher than that for the other regions, we anticipated better classification performance for this class compared to the tumor and peritumor regions. Specifically, the ratio of healthy skin pixels to tumor pixels was approximately 40:1, while the ratio to peritumor pixels was about 11:1.

Class III achieved the highest F1 score, indicating a strong agreement between precision and recall for healthy skin regions. Balanced accuracy, which considers sensitivity and specificity, exhibited trends similar to the overall accuracy across all scenarios. To ensure a fair evaluation of classifier performance, class balance was maintained throughout the training and testing phases.

In scenarios utilizing both RGB and tissue index images, the classifier demonstrated high recall for Class III, indicating the accurate identification of healthy skin regions. This performance aligns with the distinctive features captured by the tissue index images, particularly the greater non-uniformity seen in healthy skin compared to tumor and peritumor regions, which enhances the classifier’s effectiveness for Class III lesions.

Distinguishing between Class I and Class II lesions proved more challenging, especially when only RGB images were used. Nevertheless, the classifiers maintained high accuracy, precision, recall, F1 score, and balanced accuracy across all classes.

The differing performance across scenarios emphasizes the importance of the input data in classification tasks. As shown in Figure 6, Scenario I displayed substantial variation with RGB images, indicating potential reliability issues. In contrast, the stability observed in Scenario II, with tissue index images, suggests that leveraging these indices can enhance classifier performance. This improvement results from the comprehensive spectral information captured by tissue index images, which provide valuable physiological insights—reflecting properties like oxygenation levels, water content, and melanin distribution—that help distinguish healthy tissues from diseased ones. Moreover, tissue index images capture texture and morphology features specific to various tissue types, contributing to the classifier’s stability and providing reliable indicators across multiple assessments.

In comparison to the recent study by Parasca et al. [17], who utilized HSI primarily through SAM for assessing skin carcinoma margins, our research adopted a fundamentally different approach. While, in their study, the authors segmented hyperspectral images based on similar spectral properties, with notable AUC values above 0.89 for distinguishing various tissue types, we focused on utilizing biologically significant tissue indices that reflect morphological and physiological changes related to malignancy.

Methodologically, Parasca et al. [17] employed a single classifier in their analysis, whereas our approach involves an integrated pipeline that combines feature extraction, feature selection, and classification. This framework allows for flexibility and adaptability in classifying different types of skin lesions, enabling the selection of optimal classifiers for better performance. While our current study focused on malignant lesions, this approach has the potential to be extended to classify benign and other types of skin lesions, indicating its broader applicability.

In our study, the mean AUC for tumor versus the rest and peritumor versus the rest reached 0.96 and 0.94, respectively, when tissue indices were incorporated into the analysis. While these findings demonstrate the strong discriminative ability of our approach, they are preliminary, and further validation on a larger dataset is required. Overall, both studies highlight the potential of HSI for skin lesion classification; however, our work emphasizes the importance of integrating features extracted from tissue index images to improve classification accuracy and interpretability.

In comparison to the study by Lindholm et al. [14], who faced challenges in accurately classifying BCC due to ill-defined tumor borders and the misclassification of surrounding healthy skin as lesions, our research integrated the classification of peritumor regions. While Lindholm et al. [14] achieved promising sensitivity and specificity rates for differentiating lesions, our approach emphasizes the importance of considering this surrounding tissue in the overall analysis of skin lesions.

While Courtenay et al. [18] utilized near-infrared hyperspectral imaging in the range of 900.6 to 1454.8 nm and achieved AUPRC values of 0.78 for differentiating healthy skin from BCC and 0.75 for healthy skin versus SCC, our research employed a different spectral range of 400 to 1000 nm. From this range, tissue index images were calculated using specific spectral bands. The AUC values in our analysis indicated that the tissue indices demonstrated higher discriminative power in distinguishing healthy tissues from BCC and SCC, ultimately leading to improved classification accuracy.

It is essential to acknowledge certain limitations of our study. The relatively small dataset size of 24 skin lesions of skin carcinomas in the head and neck region may have influenced our results, potentially limiting the generalizability of our findings. Factors contributing to this small dataset included strict inclusion/exclusion criteria, limited consent from eligible patients, and challenges with data acquisition during the post-COVID-19 period.

We recognize the potential for overfitting when selecting seven radiomic features out of a large pool of features from a dataset consisting of 72 data points. For each of these data points, we analyzed 12 tissue index images and their corresponding RGB images. The selected textural features—along with a few first-order statistics—align with the features reported in previous studies [19], even though the imaging modalities differ. As noted by [19], despite the methodological variability and different feature sets utilized in prior studies, there remains consistency in the type of information provided, particularly in terms of the textural characteristics of lesions. This similarity in feature types indicates their potential prognostic value; however, we remain cautious about the bias introduced during the selection process.

Through experiments with varying numbers of features, we aimed to achieve a balance between maintaining discriminative power and recognizing the risk of overfitting, ultimately opting for seven features that ensured both statistical relevance and interpretability without losing critical information from the tissue-based features.

To evaluate model performance, we applied stratified k-fold cross-validation and used a separate test set, emphasizing that there was no data leakage at any stage of the testing phase. Additionally, the use of tissue index images derived from hyperspectral data contributed to initial dimensionality reduction and noise minimization, improving analytical robustness and reducing bias. However, despite the steps taken, there remained the possibility that the performance metrics could have been affected by biases associated with the selected features and the limited sample size. This will be addressed in future research by expanding the dataset to include new measurements of both malignant and benign skin lesions.

Moreover, due to dataset constraints, we opted not to employ deep learning methods, which often require larger datasets to train complex models effectively. While deep learning approaches have shown promise in various medical imaging tasks, including skin lesion classification, their application in this study was deemed impractical given the dataset constraints. We also investigated the potential of pretrained neural networks, including U-Net, for our classification task. However, our attempts with these models did not yield satisfactory results. This highlights the importance of dataset-specific considerations when selecting and adapting ML models. Nevertheless, future studies with larger datasets could explore the feasibility and potential benefits of incorporating deep learning methodologies to improve classification accuracy and model interpretability further.

In summary, our preliminary results indicate potential, as evidenced by the AUC values achieved in our analyses, laying the groundwork for future research aimed at integrating classification-based segmentation techniques for skin lesions, with the ultimate goal of developing a fast and non-invasive method that can discriminate between different skin lesions based on easily understandable biological features and could be applied in clinical settings. While the current study focused on the classification of skin lesions, by incorporating classification-based segmentation using radiomic features extracted from tissue index images, we aim to achieve more-precise identification and delineation of lesion boundaries in the future.

Additionally, subsequent research will involve comparing the performance of this classification-based segmentation approach with segmentation performed by deep learning networks. This comparative analysis will provide valuable insights into the strengths and limitations of different segmentation methodologies, further informing the development of robust and accurate skin lesion analysis techniques.

## 5. Conclusions

The integration of HSI with ML classifiers offers a promising direction for advancing skin lesion analysis, with a particular focus on head and neck skin carcinomas. Our study showcases the potential of using HSI as a non-invasive technique for characterizing tissues and improving tumor classification accuracy, surpassing the limitations of conventional RGB imaging alone.

An essential aspect of our work was the selection of the relevant features for classification. By narrowing down the radiomic features extracted from RGB images and tissue index images, we identified the most significant ones for distinguishing between tumor, peritumor, and healthy skin regions. Among the tissue indices, the concentration of water, erythema, and oxygenation were key contributors to accurate classification, highlighting the crucial role of optimizing model performance through careful feature selection from these indices.

The non-invasive nature of our approach and the identification of imaging biomarkers position this methodology as a valuable tool for improving skin lesion analysis.

## Figures and Tables

**Table 1:** Summary of studies utilizing hyperspectral imaging for skin lesion analysis. AUC = Area Under the Curve. AUPRC = Area Under the Precision–Recall Curve.

Study	Methodology	Sensitivity (%)	Specificity (%)	Limitations
Huang et al. [12]	Utilized AI and HSI for automatic segmentation and classification of skin lesions, employing U-Net Attention models and XGBoost.	90.72	96.76	Narrow focus on mycosis fungoides—potential interference from similar skin conditions; limited sample size; limited skin phototype diversity; uneven lighting.
Nagaoka et al. [13]	Developed a hyperspectral melanoma screening system using a melanoma discrimination index based on pigment characteristics.	90	84	Limited melanoma cases; focused on a narrow selection of lesions; limited ethnic representation.
Lindholm et al. [14]	Developed a hyperspectral melanoma screening system using hand-held SICSURFIS imager and CNN for analysis.	87 (pig.) 79 (non-pig.)	93 (pig.) 91 (non-pig)	Limited sample size; challenges in classifying BCCs due to ill-defined tumor borders; insufficient training on actinic keratoses and in situ carcinomas.
Leon et al. [15]	Developed an HSI system for pixel segmentation and classification using a combination of unsupervised and supervised algorithms.	88	100	Limited sample size; low spatial resolution of the HS camera; challenges in achieving real-time processing; need for diverse skin types and lesions.
Hosking et al. [16]	Employed multiple classification algorithms using a 21-wavelength mAID to capture HS images of pigmented lesions.	100	36	Small sample size; artificially high melanoma incidence.
Parasca et al. [17]	Utilized HSI to analyze tumor margins in carcinomas, employing a segmentation and classification method based on spectral features.	AUC >0.89	-	Small sample size; heterogeneity in histological subtypes.
Courtenay et al. [18]	Utilized near-infrared hyperspectral imaging and convolutional neural networks combined with support vector machines for classification of non-melanoma skin cancer and actinic keratosis.	AUPRC 0.84 (healthy vs. BCC)	AUPRC 0.80 (healthy vs. SCC)	Limited to specific demographics; focused on fair-skinned patients; need for larger, more diverse samples to improve generalizability.

**Table 2:** Tissue indices used in the analysis with corresponding symbols and descriptions.

Symbol	Name	Description	
$E_{\mathrm{Daw}}$	Dawson’s erythema index	tissue blood content estimation	[25]
$E_{c,\mathrm{Daw}}$	corrected Dawson’s erythema index	tissue blood content estimation	[25]
$E_{\mathrm{Diff}}$	Diffey’s erythema index	tissue blood content estimation	[25]
$M_{\mathrm{Daw}}$	Dawson’s melanin index	melanin content estimation	[25]
$oxy_{H}$	Huang’s oxygenation index	superficial skin oxygenation estimation	[26]
$oxy_{I}$	Ishimaru’s oxygenation index	deeper skin oxygenation estimation	[27]
$E_{\mathrm{Wag}}$	Wagner’s erythema index	tissue blood content estimation	[28]
$M_{\mathrm{Wag}}$	Wagner’s melanin index	melanin content estimation	[28]
$oxy_{T}$	oxygenation index	oxygenation estimation	[29]
TWI	water index	tissue water concentration estimation	[29]
THI	total hemoglobin index	hemoglobin estimation	[29]
NTP	NIR perfusion index	perfusion estimation	[29]

**Table 3:** Selected radiomic features for each scenario.

Feature Number	Scenario I	Scenario II	Scenario III
1	GLDM Dependence Non-Uniformity	GLCM Informational Measure of Correlation 1 $E_{c,\mathrm{Daw}}$	GLCM Informational Measure of Correlation 1 $E_{c,\mathrm{Daw}}$
2	GLRLM Gray-Level Non-Uniformity	FIRSTORDER Energy $oxy_{I}$	GLRLM Run Length Non-Uniformity RGB
3	GLRLM Run Length Non-Uniformity	FIRSTORDER Energy $oxy_{H}$	FIRSTORDER Energy $oxy_{I}$
4	GLDM Gray-Level Non-Uniformity	GLDM Dependence Non-Uniformity TWI	FIRSTORDER Energy $oxy_{H}$
5	NGTDM Busyness	GLRLM Run Length Non-Uniformity $E_{c,\mathrm{Daw}}$	GLRLM Run Length Non-Uniformity $E_{\mathrm{Diff}}$
6	NGTDM Coarseness	GLZSM Zone Percentage $E_{\mathrm{Wag}}$	GLZSM Zone Percentage $E_{\mathrm{Wag}}$
7	FIRSTORDER Energy	GLRLM Run Length Non-Uniformity $E_{\mathrm{Diff}}$	GLDM Dependence Non-Uniformity TWI

**Table 4:** Hyperparameters of classifiers generated by TPOT for each scenario.

Scenario	Classifier	Hyperparameter	Value
Scenario I	Gradient Boosting Classifier	Maximum Depth	8
		Maximum Feature Subset	0.2
		Minimum Samples per Leaf	4
		Minimum Samples to Split	5
Scenario II	Extra Trees Classifier	Bootstrap	True
		Max Features	0.25
		Minimum Samples Leaf	5
		Minimum Samples Split	20
		Random State	111
Scenario III	Stacking Estimator (SGD Classifier)	Alpha	0.0
		Eta0	0.01
		Fit Intercept	False
		L1 Ratio	0.0
		Learning Rate	’invscaling’
		Loss	’modified huber’
		Penalty	’elasticnet’
		Power T	100.0
	Zero Count	-	-
	Linear SVC Classifier	Dual	False
		Penalty	’l1’
		Tolerance	0.001

**Table 5:** Comparison of performance metrics across scenarios. This table presents the mean ± standard deviation for key performance metrics, including accuracy, precision, recall, F1 score, and balanced accuracy. Class I represents tumors, Class II represents peritumors, and Class III represents healthy skin regions. Note: The value for the mean precision for Class III was omitted in Scenario III, where a perfect score of 1.00 was achieved. The mean recall values for Class III were omitted in Scenarios I and II for the same reason. These results may have been influenced by bias from certain extracted features related to region size.

Metric	RGB Images	Tissue Index Images	Combined Scenario
Mean Accuracy	0.8773 ± 0.0186	0.9175 ± 0.0087	0.9203 ± 0.0138
Mean Precision	0.8784 ± 0.0182	0.9189 ± 0.0079	0.9236 ± 0.0130
Mean Precision [Class I]	0.8006 ± 0.0350	0.8559 ± 0.0221	0.9076 ± 0.0297
Mean Precision [Class II]	0.8399 ± 0.0217	0.9047 ± 0.0119	0.8634 ± 0.0271
Mean Precision [Class III]	0.9946 ± 0.0135	0.9960 ± 0.0120	-
Mean Recall	0.8773 ± 0.0186	0.9175 ± 0.0087	0.9203 ± 0.0138
Mean Recall [Class I]	0.8513 ± 0.0206	0.9111 ± 0.0141	0.8763 ± 0.0273
Mean Recall [Class II]	0.7805 ± 0.0492	0.8416 ± 0.0292	0.9333 ± 0.0254
Mean Recall [Class III]	-	-	0.9513 ± 0.0324
Mean F1 Score	0.8768 ± 0.0190	0.9173 ± 0.0089	0.9209 ± 0.0136
Mean F1 Score [Class I]	0.8247 ± 0.0223	0.8824 ± 0.0115	0.8912 ± 0.0199
Mean F1 Score [Class II]	0.8085 ± 0.0328	0.8717 ± 0.0154	0.8966 ± 0.0197
Mean F1 Score [Class III]	0.9972 ± 0.0069	0.9979 ± 0.0061	0.9748 ± 0.0170
Mean Balanced Accuracy	0.8773 ± 0.0186	0.9175 ± 0.0087	0.9203 ± 0.0138

## Data Availability

The data that support the findings of this study are not openly available due to sensitivity reasons and are available from the corresponding author upon reasonable request.

## Appendix A

**Table A1:** Clinical and histopathological data of the patients with BCC and SCC included in this study.

Patient	Age (Years)/Sex	Tumor Location	Histopathological Type
1	82/F	Left nasal ala	SCC
2	80/F	Nasal apex	BCC
3	60/M	Nose radix	BCC
4	83/M	Left pinna	SCC
5	95/F	Left temporal region	BCC
6	78/M	Right cheek	BCC
		Right inferior lip	BCC
7	80/M	Left pinna	BCC
		Left pinna	SCC
8	82/M	Nose apex	BCC
9	87/M	Left nasal ala	BCC
10	93/M	Nasal apex	BCC
11	74/F	Right nasal ala	BCC
12	71/F	Right nasal ala	BCC
13	88/M	Right pinna	BCC
		Left preauricular region	BCC
14	82/F	Left nasal ala	BCC
15	90/M	Left temporal region	SCC
		Left temple	Atypia
		Right supraorbital region	SCC
		Right supraorbital region	SCC
		Right frontal region	SCC
16	86/M	Right neck	BCC
		Right frontal region	SCC

## Appendix B

### Appendix B.1. Inclusion and Exclusion Criteria for Patient Selection

One week prior to inclusion in the study, we

- Obtained medical history and performed clinical examination;
- Administered EQ-5D questionnaire;
- Obtained photographic documentation of tumors (hyperspectral imaging);
- Measured tumor size;
- Ran laboratory tests: (hemogram and differential blood count, coagulation tests, biochemical tests);
- Performed an electrocardiogram (ECG);
- Conducted imaging studies (X-ray, CT, MRI) as necessary, based on the clinical situation.

Patients were selected based on inclusion and exclusion criteria.

### Appendix B.2. Inclusion Criteria

- Histologically or cytologically confirmed squamous cell or basal cell carcinoma in the head and neck area.
- Tumors (primary, regional, or systemic metastases) that were not suitable for standard treatment (surgery, radiotherapy, systemic therapy) due to
  - The patient’s general condition and/or;
  - The number of tumors (multiple tumors in different locations) and/or;
  - Tumor location in areas where we expected poor functional or aesthetic results and/or;
  - The patient’s refusal of other established forms of treatment.
- Age over 18 years.
- Expected lifespan of more than 3 months.
- Karnofsky performance status of ≥70 or ≥2 on the WHO scale.
- At least 3 months had passed since any previous treatment.
- The patient’s ability to understand the treatment procedure and potential side effects that may occur during treatment.
- The patient was capable of signing informed consent to participate in this clinical study.
- Before inclusion in this study, the patient presented at a multidisciplinary consultation.

### Appendix B.3. Exclusion Criteria

- Lesions that were not suitable for treatment with electrochemotherapy (bone invasion, infiltration of major vessels).
- Life-threatening infection and/or heart failure and/or liver failure and/or other life-threatening systemic disease states.
- Significantly reduced lung function that required measurement of lung diffusion capacity. Patients were not requiring treatment if the result of the lung diffusion capacity measurement was outside normal values.
- Age under 18 years.
- Significant coagulation disorders (that did not respond to standard therapy: vitamin K replacement or fresh frozen plasma).
- Previous received cumulative dose of bleomycin $\geq 250,000\;{\mathrm{IU}/m}^{2}$.
- Allergic reaction to prior treatment with bleomycin.
- Chronic reduction of renal function (creatinine >150μmol/L).
- Epilepsy.
- Pregnancy.
- Patients who were unable to understand the purpose or conduct of this study or did not agree to participate in this study.

## Appendix C. Equations for Calculation of Tissue Indices

### Appendix C.1. Dawson’s Melanin Index

The melanin index ($M_{\mathrm{Daw}}$) is calculated based on the difference between the average absorbance values over two spectral ranges. It is defined as follows [25]:

(A1) $$M_{\mathrm{Daw}}=\left(L_{640-650}-L_{690-700}\right)\times 100$$

where $L_{x-y}$ is the average absorption in the wavelength range from *x* to *y* nm.

### Appendix C.2. Dawson’s Erythema Index

The Dawson’s erythema index ($E_{\mathrm{Daw}}$), a parameter that is proportional to the area under the hemoglobin absorption curve when an artificial baseline is drawn between 510 nm and 610 nm, is defined as follows [25]:

(A2) $$E_{\mathrm{Daw}}=100\times \left[A_{560}+1.5\cdot \left(A_{540}+A_{575}\right)-2\cdot \left(A_{510}+A_{610}\right)\right]$$

where $A_{n}$ is the absorption of the spectrum at wavelength *n*.

### Appendix C.3. Corrected Dawson’s Erythema Index

The corrected Dawson’s erythema index ($E_{c}$) is defined as follows [25]:

(A3) $$E_{c,\mathrm{Daw}}=E_{\mathrm{Daw}}\left(1-\gamma \cdot M_{\mathrm{Daw}}\right)$$

where $E_{c}$ is corrected for melanin by adding the melanin index, which is scaled by a factor γ (where γ=0.04). The scaling factor was empirically determined, assuming that lightly and darkly pigmented subjects had a similar blood content.

### Appendix C.4. Diffey’s Erythema Index

Diffey’s erythema index ($E_{\mathrm{dif}}$) is defined as follows [25]:

(A4) $$E_{\mathrm{Diff}}=5\log_{10}\left(\frac{I_{635}}{I_{565}}\right)$$

where $I_{x}$ is the reflectance at wavelength *x* nm.

### Appendix C.5. Huang’s Oxygenation Index

Huang’s oxygenation index ($oxy_{H}$) is calculated as follows [26]:

(A5) $$oxy_{H}=100\left(-1.4\cdot {\mathrm{SDR}}^{3}+4.82\cdot {\mathrm{SDR}}^{2}-5.66\cdot \mathrm{SDR}+2.38\right)$$

where SDR stands for the second derivative ratio of *r* (where $r=\frac{\mu_{a}}{\mu_{s}^{\prime }}$), which is the ratio between the absorption coefficient and the scattering coefficient. The second derivative ratio (SDR) is calculated using the following equation:

$$\mathrm{SDR}=\frac{I_{592}+I_{544}-2\cdot I_{568}}{I_{600}+I_{552}-2\cdot I_{576}}$$

where $I_{x}$ is the reflectance at the given wavelengths.

### Appendix C.6. Ishimaru’s Oxygenation Index

Ishimaru’s oxygenation index ($oxy_{I}$) is calculated as follows [27]:

(A6) $$oxy_{I}=100\cdot \frac{9.8860-4.3299\left(\frac{I_{920}}{I_{660}}\right)}{9.8860-1.0434}$$

where $I_{x}$ represents the reflectance at the given wavelengths.

### Appendix C.7. Wagner’s Melanin Index

Wagner’s melanin index ($M_{\mathrm{Wag}}$) is calculated using the following equation [28]:

(A7) $$M_{\mathrm{Wag}}=-100\cdot \log\left(\mathrm{Eqn}\;1\right)$$

where

$$\mathrm{Eqn}\;1=\frac{I_{650}+I_{660}+0.5\cdot I_{640}+0.5\cdot I_{670}}{3}$$

and $I_{x}$ represents the reflectance at the given wavelengths.

### Appendix C.8. Wagner’s Erythema Index

Wagner’s erythema index ($E_{\mathrm{Wag}}$) is given by [28]:

(A8) $$E_{\mathrm{Wag}}=-100\cdot \log\left(\mathrm{Eqn}\;3\right)-M_{\mathrm{Wag}}$$

where

$$\mathrm{Eqn}\;3=\frac{0.5\cdot I_{560}+I_{570}+0.5\cdot I_{580}}{2}$$

and $I_{x}$ represents the reflectance at the given wavelengths, and $M_{\mathrm{Wag}}$ is the Wagner’s melanin index calculated previously.

### Appendix C.9. Oxygenation Index (TIVITA)

The oxygenation index is calculated using the following equation [29]:

(A9) $$oxy_{T}=\frac{\min\left(A_{570\ldots 590}^{\prime \prime }\right)/r_{1}}{\min\left(A_{570\ldots 590}^{\prime \prime }\right)/r_{1}+\min\left(A_{740\ldots 780}^{\prime \prime }\right)/r_{2}}$$

where

- $A^{\prime \prime }$ represents the second derivative of the absorbance spectrum.
- The second derivative is calculated in two spectral regions where changes in oxygenated and deoxygenated hemoglobin can be observed.
- The first region is between 570 nm and 590 nm, where the significant peak structures of oxyhemoglobin are present.
- The second region is between 740 nm and 780 nm, where a distinct peak of deoxyhemoglobin is visible.
- $r_{1}$ and $r_{2}$ are scaling factors used to calibrate the resulting values based onthe reference measurements obtained with a tissue oximeter.

### Appendix C.10. NIR Perfusion Index

The NIR perfusion index (*v*) is calculated using the following equation [29]:

(A10) $$v=\frac{\mathrm{mean}{\left(A\right)}_{\left[825\;–\;925\;\mathrm{nm}\right]}}{\mathrm{mean}{\left(A\right)}_{\left[655\;–\;735\;\mathrm{nm}\right]}}-s_{1}/\left(s_{2}-s_{1}\right)$$

where

- $\mathrm{mean}{\left(A\right)}_{\left[825\;–\;925\;\mathrm{nm}\right]}$ is the mean absorbance calculated over the wavelength range from 825 to 925 nm.
- $\mathrm{mean}{\left(A\right)}_{\left[655\;–\;735\;\mathrm{nm}\right]}$ is the mean absorbance calculated over the wavelength range from 655 to 735 nm.
- The scaling parameters $s_{1}$ and $s_{2}$ are used to calibrate the resulting values to fit the range from 0 to 100.

### Appendix C.11. Tissue Hemoglobin Index (THI)

The calculation of the Tissue Hemoglobin Index [29] is carried out using the same equation as the NIR perfusion index (Equation A10), with the specified spectral ranges and individual scaling variables. In this case, the analysis is conducted within the spectral ranges of 530–590 nm and 785–825 nm.

### Appendix C.12. Tissue Water Index (TWI)

The calculation of the Tissue Water Index [29] follows the same method as that used for the Tissue Hemoglobin Index (THI) and the NIR perfusion index, but the analysis is conducted within the spectral ranges of 880–900 nm and 955–980 nm.

## References

[1] Urban K., Mehrmal S., Uppal P., Giesey R.L., Delost G.R. (2021). The global burden of skin cancer: A longitudinal analysis from the Global Burden of Disease Study, 1990-2017. JAAD Int..

[2] Papageorgiou V., Apalla Z., Sotiriou E., Papageorgiou C., Lazaridou E., Vakirlis S., Ioannides D., Lallas A. (2018). The limitations of dermoscopy: False-positive and false-negative tumours. J. Eur. Acad. Dermatol. Venereol..

[3] Kye S., Lee O. (2024). Hyperspectral imaging-based erythema classification in atopic dermatitis. Ski. Res. Technol..

[4] Lu G., Fei B. (2014). Medical Hyperspectral Imaging: A Review. J. Biomed. Opt..

[5] Ortega S., Halicek M., Fabelo H., Callico G.M., Fei B. (2020). Hyperspectral and multispectral imaging in digital and computational pathology: A systematic review. Biomed. Opt. Express.

[6] Hren R., Sersa G., Simoncic U., Milanic M. (2022). Imaging perfusion changes in oncological clinical applications by hyperspectral imaging: A literature review. Radiol. Oncol..

[7] Shapey J., Xie Y., Nabavi E., Bradford R., Saeed R., Ourselin S., Vercauteren T. (2019). Intraoperative multispectral and hyperspectral label-free imaging: A systematic review of in vivo clinical studies. J. Biophoton..

[8] Zhang Y., Wu X., He L., Meng C., Du S., Bao J., Zheng Y. (2020). Applications of hyperspectral imaging in the detection and diagnosis of solid tumors. Transl. Cancer Res..

[9] Yoon J. (2022). Hyperspectral Imaging for Clinical Applications. BioChip J..

[10] Aloupogianni E., Ishikawa M., Kobayashi N., Obi T. (2022). Hyperspectral and multispectral image processing for gross-level tumor detection in skin lesions: A systematic review. J. Biomed. Opt..

[11] Mangotra H., Srivastava S., Jaiswal G., Rani R., Sharma A. (2023). Hyperspectral imaging for early diagnosis of diseases: A review. Expert Syst..

[12] Huang H.Y., Nguyen H.T., Lin T.L., Saenprasarn P., Liu P.H., Wang H.C. (2024). Identification of Skin Lesions by Snapshot Hyperspectral Imaging. Cancers.

[13] Nagaoka T., Nakamura A., Okutani H., Kiyohara Y., Sota T. (2012). A possible melanoma discrimination index based on hyperspectral data: A pilot study. Ski. Res. Technol..

[14] Lindholm V., Raita-Hakola A.M., Annala L., Salmivuori M., Jeskanen L., Saari H., Koskenmies S., Pitkänen S., Pölönen I., Isoherranen K. (2022). Differentiating Malignant from Benign Pigmented or Non-Pigmented Skin Tumours—A Pilot Study on 3D Hyperspectral Imaging of Complex Skin Surfaces and Convolutional Neural Networks. J. Clin. Med..

[15] Leon R., Martinez-Vega B., Fabelo H., Ortega S., Melian V., Castaño I., Carretero G., Almeida P., Garcia A., Quevedo E. (2020). Non-Invasive Skin Cancer Diagnosis Using Hyperspectral Imaging for In-Situ Clinical Support. J. Clin. Med..

[16] Hosking A.M., Coakley B.J., Chang D., Talebi-Liasi F., Lish S., Lee S.W., Zong A.M., Moore I., Browning J., Jacques S.L. (2019). Hyperspectral imaging in automated digital dermoscopy screening for melanoma. Lasers Surg. Med..

[17] Parasca S.V., Calin M.A., Manea D., Radvan R. (2024). Hyperspectral imaging with machine learning for in vivo skin carcinoma margin assessment: A preliminary study. Phys. Eng. Sci. Med..

[18] Courtenay L.A., Barbero-García I., Martínez-Lastras S., Del Pozo S., Corral M., González-Aguilera D. (2024). Using computational learning for non-melanoma skin cancer and actinic keratosis near-infrared hyperspectral signature classification. Photodiagnosis Photodyn. Ther..

[19] Corti A., Cavalieri S., Calareso G., Mattavelli D., Ravanelli M., Poli T., Licitra L., Corino V.D.A., Mainardi L. (2024). MRI radiomics in head and neck cancer from reproducibility to combined approaches. Sci. Rep..

[20] McCulloch P., Cook J.A., Altman D.G., Heneghan C., Diener M.K., Group I. (2013). IDEAL framework for surgical innovation 1: The idea and development stages. BMJ (Clin. Res. Ed.).

[21] Bjorgan A., Randeberg L.L. (2015). Real-Time Noise Removal for Line-Scanning Hyperspectral Devices Using a Minimum Noise Fraction-Based Approach. Sensors.

[22] Péus D., Newcomb N., Hofer S. (2013). Appraisal of the Karnofsky Performance Status and proposal of a simple algorithmic system for its evaluation. BMC Med. Inform. Decis. Mak..

[23] Smith T., Guild J. (1931). The C.I.E. colorimetric standards and their use. Trans. Opt. Soc..

[24] Stergar J., Lakota K., Perše M., Tomšič M., Milanič M. (2022). Hyperspectral evaluation of vasculature in induced peritonitis mouse models. Biomed. Opt. Express.

[25] Riordan B., Sprigle S., Linden M. (2001). Testing the validity of erythema detection algorithms. J. Rehabil. Res. Dev..

[26] Huang J. (2012). Multispectral Imaging of Skin Oxygenation. Ph.D. Thesis.

[27] Ishimaru A. (1978). Wave Propagation and Scattering in Random Media.

[28] Wagner J.K., Jovel C., Norton H.L., Parra E.J., Shriver M.D. (2002). Comparing Quantitative Measures of Erythema, Pigmentation and Skin Response using Reflectometry. Pigment Cell Research.

[29] Holmer A., Marotz J., Wahl P., Dau M., Kämmerer P.W. (2018). Hyperspectral imaging in perfusion and wound diagnostics—Methods and algorithms for the determination of tissue parameters. Biomed. Eng./Biomed. Tech..

[30] Kruse F., Lefkoff A., Boardman J., Heidebrecht K., Shapiro A., Barloon P., Goetz A. (1993). The spectral image processing system (SIPS)—Interactive visualization and analysis of imaging spectrometer data. Remote Sens. Environ..

[31] van Griethuysen J.J., Fedorov A., Parmar C., Hosny A., Aucoin N., Narayan V., Beets-Tan R.G., Fillion-Robin J.C., Pieper S., Aerts H.J. (2017). Computational Radiomics System to Decode the Radiographic Phenotype. Cancer Res..

[32] Scapicchio C., Gabelloni M., Barucci A., Cioni D., Saba L., Neri E. (2021). A deep look into radiomics. Radiol. Medica.

[33] Ding C., Peng H. Minimum redundancy feature selection from microarray gene expression data. Proceedings of the Computational Systems Bioinformatics (CSB2003).

[34] Le T.T., Fu W., Moore J.H. (2020). Scaling tree-based automated machine learning to biomedical big data with a feature set selector. Bioinformatics.

[35] Olson R.S., Urbanowicz R.J., Andrews P.C., Lavender N.A., Kidd L.C., Moore J.H. (2016). Automating biomedical data science through tree-based pipeline optimization. Proceedings of the 19th European Conference of the Applications of Evolutionary Computation (EvoApplications 2016).

[36] Olson R.S., Bartley N., Urbanowicz R.J., Moore J.H. Evaluation of a Tree-based Pipeline Optimization Tool for Automating Data Science. Proceedings of the Genetic and Evolutionary Computation Conference 2016 (GECCO ’16).

[37] Arnold C., Biedebach L., Küpfer A., Neunhoeffer M. (2024). The role of hyperparameters in machine learning models and how to tune them. Political Sci. Res. Methods.

[38] Pedregosa F., Varoquaux G., Gramfort A., Michel V., Thirion B., Grisel O., Blondel M., Prettenhofer P., Weiss R., Dubourg V. (2011). Scikit-learn: Machine Learning in Python. J. Mach. Learn. Res..

[39] Jeon S., Jeon M., Choi S., Yoo S., Park S., Lee M., Kim I. (2023). Hypoxia in Skin Cancer: Molecular Basis and Clinical Implications. Int. J. Mol. Sci..

[40] Jeong J.H., Ojha U., Lee Y.M. (2021). Pathological angiogenesis and inflammation in tissues. Arch. Pharmacal Res..

